# Antithyroid Drugs

**DOI:** 10.22037/ijpr.2020.112892.14005

**Published:** 2019

**Authors:** Hengameh Abdi, Atieh Amouzegar, Fereidoun Azizi

**Affiliations:** *Endocrine Research Center, Research Institute for Endocrine Sciences, Shahid Beheshti University of Medical Sciences, Tehran, Iran.*

**Keywords:** Antithyroid, Thionamide, Methimazole, Propylthiouracil, Graves, Hyperthyroidism

## Abstract

The thionamide drugs, *i.e.* carbimazole and its metabolite methimazole (MMI), and propylthiouracil (PTU) have extensively been used in the management of various forms of hyperthyroidism over the past eight decades. This review aims to summarize different aspects of these outstanding medications. Thionamides have shown their own acceptable efficacy and even safety profiles in treatment of hyperthyroidism, especially GD in both children and adults and also during pregnancy and lactation. Of the antithyroid drugs (ATDs) available, MMI is the preferred choice in most situations taking into account its better efficacy and less adverse effects accompanied by once-daily dose prescription because of a long half-life and similar cost. Considering the more severe teratogenic effects of MMI, PTU would be the selected ATD for treatment of hyperthyroidism during pre-pregnancy months and the first 16 weeks of gestation. Recent studies have confirmed the efficacy and safety of long-term MMI therapy with low maintenance doses for GD and toxic multinodular goiter. Despite the long-term history of ATD use, there is still ongoing debate regarding their pharmacology and diverse mechanisms of action, viz. their immunomodulatory effects, and mechanisms and susceptibility factors to their adverse reactions.

## Introduction

Hyperthyroidism, a common thyroid disorder, refers to increased synthesis and secretion of thyroid hormones by the thyroid gland. Most common causes of hyperthyroidism are Graves’ disease (GD), toxic multinodular goiter and toxic adenoma. There are three therapeutic choices for hyperthyroidism: thyroid surgery as the oldest modality, radioactive iodine (RAI) and antithyroid drugs (ATDs) (1). 

**Figure 1:**
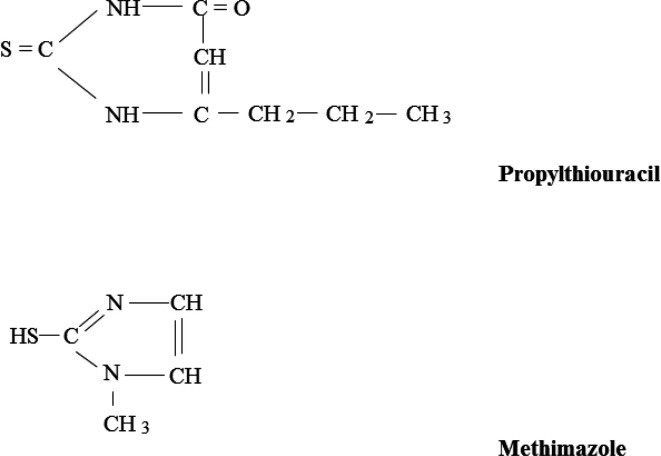
The structure of propylthiouracil and methimazole

More than 75 years ago, the inhibitory effects of thioureas/thiouracils on thyroid function were documented in animal studies (2, 3), with the first successful use of these agents in hyperthyroid patients being reported 1-2 years later (4, 5). Not too long after, the US FDA approval for thioureylene-containing antithyroid medications was issued for propylthiouracil (PTU), methimazole (MMI), and carbimazole, respectively (Figure 1). Since then, ATDs have consistently been used for treatment of various etiologies of hyperthyroidism. Compared to RAI, ATDs have almost always been the preferred therapeutic modality for GD worldwide (6-8) except in North America where remarkable change of the practice pattern in treatment of GD, *i.e.* more trend toward ATDs, has recently been documented (9). Considering the widespread use of ATDs and recent data about their long-term efficacy and safety, this review aims to summarize different aspects of these outstanding medications, focusing on the most recent evidence regarding their clinical utility.

**Mechanism of action**

**Figure 2:**
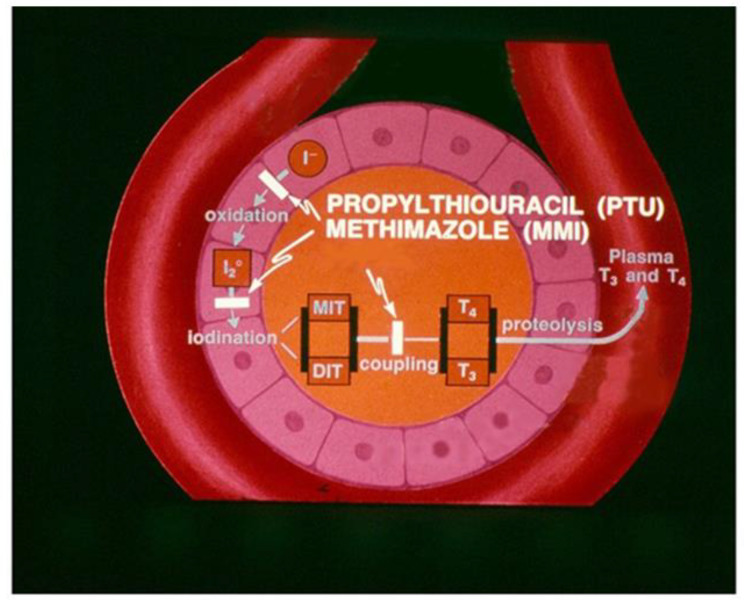
Schematic diagram of a thyroid follicle showing the main intrathyroidal actions of two thionamides, propylthiouracil and methimazole

The main mechanism of intrathyroidal action of ATDs for inhibition of thyroid hormone synthesis is competition with thyroglobulin tyrosine residues for thyroid peroxidase (TPO)-catalyzed iodination, *i.e.* iodine organification, thereby decreasing numbers of mono- and di-iodotyrosines; they also interfere with the TPO-catalyzed coupling of iodotyrosines to form thyroid hormones, 3,5,3′-triiodothyronine (T3) and tetraiodothyronine (T4) (Figure 2) (10, 11). Other inhibitory effects of ATDs proposed at the late 1970s (12, 13) need to be investigated further.

It is known that PTU inhibits the type 1 deiodinase-catalyzed T4 to T3 conversion in both the thyroid and peripheral tissues (14); this metabolic pathway contributes to nearly one-fifth of serum T3 in normal physiology and at least half in the setting of hyperthyroidism (11).

An interesting aspect of ATDs properties, not yet fully recognized, is their effects on the immune system with remarkable *in-vitro* evidence but questionable *in-vivo* findings regarding the issue whether the observed immunosuppressive effect is the direct ATD property or the consequence of decreased thyroid hormone levels (11, 15). Immunomodulatory effects of ATDs on both humoral and cell-mediated immunity have been investigated (16-20).

Evidence regarding the antioxidant properties of ATDs is scarce and contradictory (21-23).

**Clinical pharmacology**

Pharmacokinetic and pharmacodynamic properties of ATDs have been summarized in Table 1. MMI has a long serum and intrathyroidal half-life (24). Based on *in-vitro* findings, there is no significant difference between potency of MMI and PTU for inhibition of TPO-catalyzed reactions; hence, the well-known more potent antithyroid action of MMI compared to PTU, even up to 50 times (25), is related to differences in their intrathyroidal metabolism (11, 26). Data on peripheral metabolism of ATDs are scarce; a substantial portion of ATDs and their metabolites is excreted in the bile; however, their fecal excretion is very low due to the enterohepatic circulation. Small amounts of ATDs and their metabolites are detected in urine (27, 28).

**Clinical use**

Clinical conditions in which ATDs can be used as a transient or long-term therapeutic option include GD, toxic multinodular goiter, and preparation for radioactive iodine (RAI) therapy or surgery in thyrotoxic patients who are elderly or have cardiovascular disease or severe hyperthyroidism. Compared to RAI therapy and surgery, ATDs are the most favorable treatment modality in GD and usually the least selected modality in TMNG and toxic adenoma in particular (1); however, a recent RCT from Iran has documented non-inferiority and safety of long-term MMI therapy compared to RAI in 107 patients with TMNG (35).

Considering the better efficacy and less adverse effects of MMI accompanied by its once-daily dose prescription because of its long half-life and similar cost (36), this thionamide is the preferred ATD recommended by the American Thyroid Association (ATA) task force. PTU is the selected ATD for exceptional conditions, including pre-pregnancy months and the first trimester of pregnancy, thyroid storm, and in patients with minor adverse reactions to MMI (1, 37 and 38).

There are two strategies for ATD therapy: 1) the generally recommended approach, the titration regimen as monotherapy with ATD and 2) the block and replace regimen *i.e.* high-dose ATD combined with levothyroxine which is associated with a higher rate of ATD adverse reactions (39, 40).

The starting dose of MMI in the range of 5-40 mg in 1-2 divided doses or PTU 100-800 mg in 2-3 divided doses depends on the severity of thyrotoxicosis considering initial free T4 and total T3 levels, patient symptoms, and goiter grade (1, 15). The primary goal is rapid restoration of euthyroidism with minimal adverse drug reactions. The ATD dose can be down-titrated to maintenance daily doses of 2.5-10 mg MMI or 50-150 mg PTU over several months (11). ATD dose adjustment should be performed according to results of periodic clinical and biochemical monitoring. During early months of therapy, serum free T4 and total T3 are appropriate biochemical markers which should be assessed within 2-6 weeks after the ATD initiation. If euthyroidism is obtained, the ATD dose can be reduced by 30-50% and after reaching the minimum dose required for maintenance of the euthyroid condition, intervals of monitoring can be increased to every 2-3 months and even every 6 months in the setting of long-term therapy (1, 11).

In children and adolescents with GD, MMI is the preferred ATD with a daily dose in the range of 0.1-1 mg/kg. The ATA recommended approach for MMI prescription in patients aged ≤ 18 years is as follows: birth-1 year, 1.25 mg daily; 1-5 years, 2.5-5 mg daily; 5-10 years, 5-10 mg daily; and 10-18 years, 10-20 mg daily (1). ATDs do not require any dose adjustment in renal or liver diseases (31, 32).

**ATDs and remission of Graves’ disease**

Remission from GD is generally defined as normal thyroid function for at least 1 year after the ATD discontinuation (1). Since many years ago, investigators have been interested to discover predicting factors of the outcome of GD after ATD therapy (41) and several consistent (severity of thyrotoxicosis, goiter size, serum TRAb levels) and inconsistent (male sex, younger age, smoking, presence of orbitopathy, coexistence of psychiatric problems) risk factors have been reported (15, 37 and 42). In a recent meta-analysis, Struja *et al.,* pooled data of 54 studies with low to moderate quality and showed that pretreatment orbitopathy, smoking, thyroid volume estimated by sonography, goiter size, free T4, total T3, TRAb and TSH-binding inhibiting immunoglobulins (TBII) have significant positive associations with relapse rate of GD after withdrawal of ATD (43). Moreover, a new predictive score for recurrence of GD before the ATD initiation, the GREAT score (44), has been developed based on age, serum free T4 concentration, serum TBII level, and goiter size which needs to be validated in different populations worldwide.

**Duration of ATD therapy in Graves’ disease**

According to the ATA and ETA guidelines (1, 45), the standard duration of ATD therapy for GD is 12-18 months. Relapse rate of hyperthyroidism after the conventional duration of ATD treatment ranges from 30% to 70% (46-48). A network meta-analysis of 8 studies including 1 randomized trial and 7 cohort studies showed significantly higher relapse rates with ATD (almost 53%) compared to the RAI (15%) and surgery (10%) (49). The 2010 Cochrane review analyzed data from 4 trials with 445 patients (50-53), comparing the effect of duration of both regimens of ATD therapy on the relapse rate of GD. Six-month therapy was significantly associated with higher relapse rate compared to the 18-month duration; however, there was no significant difference between < 18-months (50%) and > 18-months (44%) of ATD treatment.

A recent meta-analysis including 6 studies with ATD duration of > 24 months in children (54-56) and adults (52, 57 and 58) with GD documented a remission rate of 57% (95% CI: 45-68%) accompanied by adverse reactions in 19% of patients, of which only 1.5% was related to major side effects. Each 1-year treatment induced 16% remission rate (59). Several studies have documented the efficacy and safety of continuous long-term treatment with ATDs beyond the traditional 12-18 months in adults and children with GD associated with significantly higher remission rate compared to short-term treatment (56, 57 and 60-64), findings which have led to development of a new paradigm for treatment of GD suggested by some experts (42) with the aim of attaining cure for Graves’ hyperthyroidism.

**ATDs and Graves’ orbitopathy**

Graves’ orbitopathy (GO) is an autoimmune disorder which presents in nearly one-third of patients with GD to some degree (65). Based on results of a recent meta-analysis including 3 high-quality RCTs comparing the effect of ATDs versus RAI on the course of GO in patients with GD, RAI therapy was associated with an odds of 3.06 (95% CI: 2.01-4.66) for development or worsening of GO (66), a finding also confirmed in a Cochrane review (67). One of clinical situations considers in decision making to select the therapeutic modality of GD is the status of GO; in the presence of active, moderate to severe GO, ATDs and surgery are the only recommended options (1). Regarding comparison of the effect of ATDs versus thyroidectomy on GO, we are awaiting results of an Austrian trial conducted among patients with moderate to severe GO (67).

**Adverse effects of ATDs**

Concerns regarding adverse effects of ATDs might influence the therapeutic modality selected for the hyperthyroid patient. Overall, 13% of Graves’ patients treated with ATDs (MMI: ~15%; PTU: ~7%) are affected by a drug side effect (49). Dose relevancy of ATD adverse effects seems to be more prominent for MMI (36). There are two types of adverse reactions: 1) the more common, minor, mostly allergic reactions, and 2) rare, major toxic reactions. Table 2 summarizes these side effects. 

Regarding management of skin reactions, there are three approaches: a) concurrent ATD and antihistamine therapy in the case of minor manifestations, b) switching to the other ATD, keeping in mind that cross-reactivity up to 50% might occur; this strategy is not recommended in settings of serious skin reactions, and c) change of the therapeutic modality to RAI or surgery (1, 36 and 45).

One of the life-threatening, fortunately rare (<0.5%) side effects of ATDs is agranulocytosis. It is recommended to obtain a baseline cell blood count before initiation of ATD because mild granulocytopenia is commonly seen in patients with untreated GD, those on ATD treatment and normal African Americans; the ATA task force questions the onset of ATD therapy in cases with absolute neutrophil count (ANC) < 1000/mm^3 ^(1). Verbal and written information about this serious adverse event and its associated symptoms should be provided for the patient. There is still no consensus regarding routine cell count monitoring because of the abrupt onset of agranulocytosis but most expert opinions are against it (1, 45). Whenever fever, and/or pharyngitis develop in patients on ATD, a prompt differential blood count should be obtained and ATD be discontinued if granulocytopenia is confirmed; moreover, broad spectrum antibiotic and the granulocyte colony stimulating factor should be prescribed in symptomatic patients (69). In the setting of agranulocytosis induced by one ATD, switching to the other ATD is contraindicated.

The second major, occasionally lethal side effect of ATDs is hepatotoxicity. Recent data from Taiwan (70) and China (71) challenges the old concept of more hepatocellular pattern of liver injury with PTU compared to more cholestatic pattern seen with MMI. Nevertheless, data analysis of 18,558 Japanese patients with GD treated by ATDs revealed that severe liver injury [alanine aminotransferase (ALT) > 8-fold or total bilirubin > 3-fold of upper limits] was significantly higher in the PTU-treated group (6.3%) compared to the MMI-treated group (1.4%). Median time to development of ATD-induced hepatic injury was 30 (range 7-314) days and no deaths were reported (72). ATA task force favors request for baseline liver enzymes and reconsideration of ATD prescription in the case of > 5-time elevation of their serum levels. It should be noted that mildly abnormal liver function tests are common (~30%) in untreated GD and within the first months of PTU therapy (73-75). Follow-up monitoring is immediately advised in patients who experience symptoms of liver injury including pruritic rash, jaundice, light-colored stool, dark urine, joint pain, nausea, abdominal discomfort, anorexia, or fatigue (1, 45); increased levels of liver enzymes > threefold of baseline values may be a harbinger of upcoming hepatic injury and an alarming signal for ATD discontinuation and weekly checks of liver function tests. Rising levels of serum alkaline phosphatase accompanied by normal liver transaminases indicate improving thyrotoxicosis with increased bone formation (76). Some experts suggest cautious use of MMI in the case of non-severe PTU-induced liver injury (1) whereas others such as the ETA task force are opposed to restarting the alternative ATD after any major side effects (45).

Other reported side effects of ATDs not mentioned in Table 2 include hypoprothrombinemia (PTU), pancreatitis (MMI) and elevated serum creatine kinase (MMI).

A recent systematic review showed that long-term (>18 months) ATD treatment in 1660 patients was associated with the occurrence of major complications in only 14 individuals; minor side effects, most commonly skin reactions, were reported in the range of 2-36% and were more prevalent with higher doses and in non-adult patients (77).

**ATDs in pregnancy and lactation**

**Figure 3:**
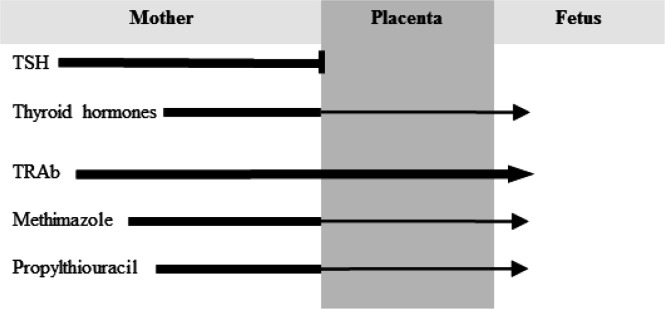
The status of passage of thyrotropin (TSH), thyroid hormones, TRAb (TSH receptor antibody) and antithyroid drugs from the maternal to fetal circulation

RAI therapy is absolutely contraindicated in pregnancy and lactation; likewise, pregnancy is a relative contraindication of thyroidectomy because of possible serious complications. Hence, ATDs with the lowest effective doses are the preferred therapy for hyperthyroidism during pregnancy (1, 45 and 78). Figure 3 depicts the status of passage of TSH, thyroid hormones, TRAb and ATDs from the maternal to fetal circulation. MMI and PTU cross the placenta equally (79, 80) and have similar efficacy regarding maintenance of the euthyroid status of the mother (81); however, based on data related to ATD risk of birth defects in the first trimester of pregnancy indicating more prevalent and more severe MMI-induced congenital malformations (82-84), PTU is the recommended ATD in the pre-pregnancy setting and during the first 16 weeks of pregnancy (1, 45 and 78). Although FDA has a black box warning against use of PTU after this period because of the risk of PTU-induced liver failure in the mother, there is no specific recommendation for the ATD type selected for the remainder duration of pregnancy by ATA task forces (1, 78). European experts consider change of PTU to MMI after 16 weeks of gestation (45). Well-documented congenital malformations associated with MMI use during early pregnancy in particular gestational age of 6-10 weeks include aplasia cutis and “MMI embryopathy” characterized by choanal and/or esophageal atresia, some dysmorphic features and development delay (11, 78 and 85). The most recent large study regarding teratogenic effects of ATDs is a nationwide Korean cohort including nearly 12,900 completed pregnancies exposed to ATD during the first trimester (86). Based on the findings of this study, fetal exposure to ATD was associated with 19% (95% CI 12-28%) increased risk of malformations. Numbers needed to harm were 91 and 46 for PTU and MMI, respectively (87). An interesting message taken from this study is that switch from MMI to PTU during early pregnancy described as exposure to both ATDs would not reduce the risk of birth defects, a finding confirmed in an older Danish cohort study by Andersen *et al.* (85). Hence, it seems logical that switch from MMI to PTU be done at the time of pregnancy planning.

In women with GD being treated with ATD who become pregnant, the need for higher doses of MMI (>5-10 mg daily) or PTU (>100-200 mg daily) to control hyperthyroid state is one predicting factor of relapse of thyrotoxicosis following ATD discontinuation (78). 

Very small amounts of the ingested dose of ATDs are concentrated in breast milk of lactating mothers: <0.08% for PTU (88) and 0.1-0.2% for MMI (31), both significantly below the therapeutic range. Several studies have shown safety of using ATDs in lactating women with regards to the child thyroid function, growth and neurodevelopmental milestone (89-92). ATA (78), ETA (45) and American Academy of Pediatrics (93) approve use of the lowest effective doses of ATDs (maximum daily dose of 20 mg for MMI and 450 mg for PTU).

## Conclusion

With regards to the natural history of most etiologies of hyperthyroidism, this common thyroid disease requires an effective and safe treatment option to be used for many years. Over the past eight decades, thionamides have shown their own acceptable efficacy and even safety profiles in management of hyperthyroidism, especially GD in both children and adults. Of the two ATDs available, MMI is the preferred drug in most situations. PTU is mostly selected for pre-pregnancy months and the first trimester of pregnancy, thyroid storm, and in patients with minor adverse reactions to MMI. ATDs can safely be prescribed in the setting of Graves’ orbitopathy. Recent studies have confirmed the efficacy and safety of long-term MMI therapy for GD and TMNG. The most common adverse effects of thionamides are minor skin reactions; their rare but major side effects *e.g.*, agranulocytosis and hepatic injury occur most likely during the first months of treatment. Despite the long-term history of ATD use, there is still ongoing debate regarding their pharmacology and diverse mechanisms of action, viz. their immunomodulatory effects, and mechanisms and susceptibility factors to their adverse reactions.

**Table 1:** Pharmacologic characteristics of antithyroid drugs.

	**Methimazole**	**Propylthiouracil**
Gastrointestinal absorption	Complete	Complete
Peak serum concentration	1-2 h after ingestion	1 h after ingestion
Volume of distribution	40 L	20 L
Protein binding	Little	80-90%
Serum half-life	6-8 h	1-2 h
Intrathyroidal concentration	5×10^-5^ mol/L	?
Clearance in liver disease	Slowed	Unchanged
Clearance in kidney disease	Unchanged	Unchanged
Duration of antithyroid action	>24 h	12-24 h
Daily dose frequency	1-2 times	3-4 times

From references (25, 29-34).

**Table 2:** Adverse reactions of antithyroid drugs

**Minor**	**Comments**
Cutaneous reactions	Most common adverse reaction; macule, urticaria, pruritus; MMI(6%) > PTU(3%); within first weeks or months of ATD initiation
Arthralgia	MMI > PTU; discontinue ATD because it may portend the major reaction of polyarthritis named antithyroid arthritis syndrome
Gastrointestinal upset	Predominantly nausea
Abnormal sense of taste	Decreased sense of taste with MMI and bitter or metallic taste with both ATDs
Sialadenitis and lymph node enlargement	Very rare
** Major**	
Agranulocytosis	Absolute granulocyte count <500/mm^3^; immune-mediated; PTU > low-dose MMI; mostly within 3 months of ATD initiation; more likely in older patients and some HLA variants
Hepatitis/cholestasis	PTU-induced hepatitis is more likely in children; MMI-induced hepatotoxicity is more common in adults aged >40 years
ANCA-positive vasculitis	A lupus-like rare syndrome; PTU > MMI; most reports from Asian countries; asymptomatic ANCA positivity may occur
Hematologic abnormalities	Pancytopenia, aplastic anemia, thrombocytopenia
Insulin autoimmune syndrome	Only with MMI; typically in Asian men; presents with hypoglycemia

From references (1, 11, 36, 45, 49 and 68).

MMI: methimazole; PTU: propylthiouracil; ATD; antithyroid drug; ANCA: antineutrophil cytoplasmic antibody.
